# Advances in Nanotechnology-Based Immunotherapy for Glioblastoma

**DOI:** 10.3389/fimmu.2022.882257

**Published:** 2022-05-16

**Authors:** Lin Tang, Ming Zhang, Chaoyong Liu

**Affiliations:** ^1^ Beijing Advanced Innovation Center for Soft Matter Science and Engineering, Beijing University of Chemical Technology, Beijing, China; ^2^ College of Life Science and Technology, Beijing University of Chemical Technology, Beijing, China; ^3^ Department of Pathology, Peking University International Hospital, Beijing, China

**Keywords:** immunotherapy, nanotechnology, blood-brain barrier, nanomaterial, glioblastoma

## Abstract

Glioblastoma (GBM) is the most aggressive type of brain tumor. Despite the multimodal therapies, the effectiveness of traditional treatments is not much satisfying. In recent years, immunotherapy has become the focus of tumor treatment. Unlike traditional treatments that directly target tumor cells, immunotherapy uses the body’s immune system to kill tumors. However, due to the severe immunosuppressive microenvironment of GBM, it generally has a poor response to immunotherapy. In addition, the existence of the blood-brain barrier (BBB) also compromises the immunotherapeutic efficacy. Therefore, effective immunotherapy of GBM requires the therapeutic agents to not only efficiently cross the BBB but also relieve the strong immunosuppression of the tumor microenvironment of GBM. In this review, we will first introduce the CNS immune system, immunosuppressive mechanism of GBM, and current GBM immunotherapy strategies. Then, we will discuss the development of nanomaterials for GBM immunotherapy based on different strategies, roughly divided into four parts: immune checkpoint therapy, targeting tumor-associated immune cells, activating immune cells through immunogenic cell death, and combination therapy, to provide new insights for future GBM immunotherapy.

## Introduction

Gliomas are believed to arise from neuroglial progenitor cells, which encompass variants of histological and molecular subtypes (1). Glioblastoma (GBM) accounts for most gliomas (58.4%) and is the most common type of all malignant central nervous system (CNS) tumors (49.1%). The incidence rate in the USA was 3.23 per 100,000 population (2). Despite the variety of therapies, including surgery, radiotherapy, and chemotherapy, the prognosis for GBM is still unsatisfactory. The median survival was 8 months and only 6.8% of patients survive for five years or more (2). All patients with GBM eventually have disease relapse.

According to the European Association for Neuro-Oncology (EANO) guidelines on the diagnosis and treatment of diffuse gliomas in adulthood, the goal of surgery is gross total resection whenever feasible, without compromising neurological function (3). GBM always has a diffuse growth pattern and infiltrates into normal brain tissue, so it is hard to attain complete resection. Radiotherapy and chemotherapy should start within 3–5 weeks after surgery, which has been part of the standard treatment for patients suffering from GBM (3, 4), alkylating agent temozolomide (TMZ) is the most used drug. However, resistance will take place during radio- and chemotherapy treatment through complex signaling pathways, including the Wingless-related integration site (Wnt), Sonic hedgehog (Shh), nuclear factor κ-light chain-enhancer of activated B cells (NF-κB), DNA damage response (DDR) enzymes, and mitogen-activated protein kinase (MAPK) pathways (5). Other chemotherapeutics like nitrosourea and bevacizumab are also approved for the treatment of progressive and recurrent GBM, but the curative effect was far from satisfactory (3, 6). Therefore, it is urgently needed to develop novel approaches to raise effective antitumor responses against GBM.

In recent years, immunotherapy has prolonged the overall survival of patients with a variety of tumors like advanced melanoma (7), non-small cell lung cancer (8), urothelial carcinoma (9), and renal cell carcinoma (10, 11). However, the therapeutic effects for GBM were not as well as the above tumors, due to the severely immunosuppressed tumor microenvironment of the GBM, and the limited penetration of the therapeutic agents across the blood-brain barrier (BBB). Therefore, developing strategies that can not only deliver the therapeutic agents efficiently across the BBB but also reverse the strong immunosuppressive microenvironment of GBM is of great significance for effective GBM immunotherapy. Most recent advances in understanding the physiology of the BBB, GBM microenvironment (GME), and the immunosuppressive mechanism of GBM have provided us with great opportunities to develop effective immunotherapeutics against GBM (12–14). The emergence of nanotechnologies provides a new development direction for the efficient targeted delivery of drugs to overcome physiological barriers and active targeting of specific cell populations, such as tumor cells/immune cell subsets. We have summarized the advances in the development of nanotechnologies to improve drug delivery across the BBB in our previous review (15). In this review, we will introduce the CNS immune system, immunosuppressive mechanism of GBM, current GBM immunotherapy, and the development of nanomaterials for GBM immunotherapy. This article will classify and summarize GBM immunotherapy based on nanomaterials, roughly divide into four parts: immune checkpoint therapy, targeting tumor-associated immune cells, activating immune cells through immunogenic cell death, and combination therapy.

## Immune microenvironment and immunotherapy strategies of glioblastoma

For a long time, the CNS was thought to be an immune-privileged system. It was evidenced by several researchers that homografts transplanted to the brain failed to elicit an immune state (16, 17). In addition, the CNS was thought to lack a classical lymphatic drainage system (18). But recent studies revealed that functional lymphatic vessels lining the dural sinuses can regulate brain tumor drainage and immunity, they can carry fluid and immune cells from the cerebrospinal fluid and are connected to the deep cervical lymph nodes, then prime T and B lymphocytes (18, 19). Furthermore, meningeal immune surveillance is critical for brain function, which is enabled by endothelial and mural cells forming the dural sinus stromal niche (20). These studies indicated that the brain is an immunological distinctive organ, but is still able to generate immune responses, which gives rise to immunotherapeutic opportunities for brain tumors.

**Figure 1:**
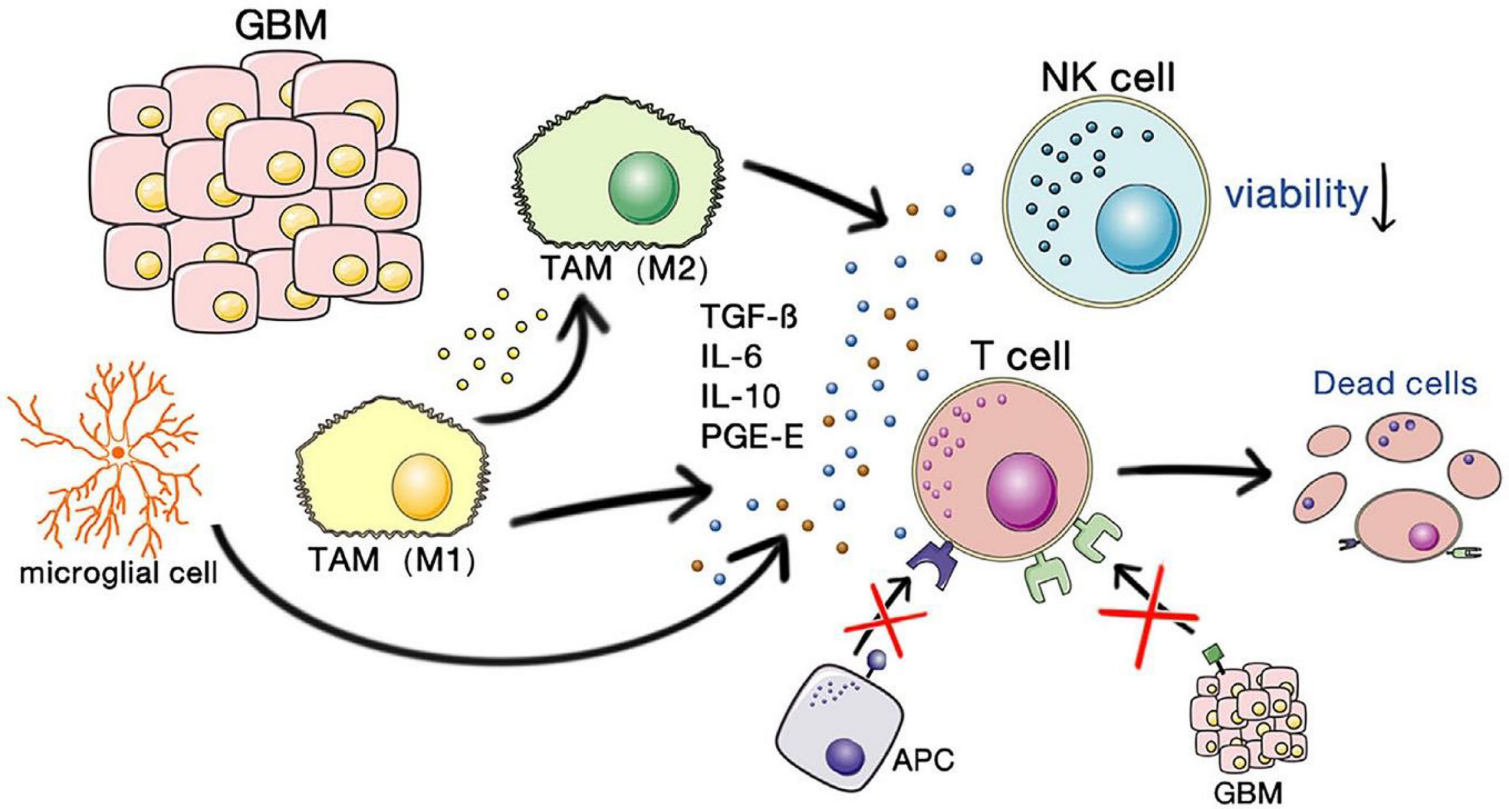
The immunosuppressive microenvironment of GBM. Copyright (24).

One of the key obstacles to effective immunotherapy of GBM lies in its highly immunosuppressive nature. The mechanisms involve both tumor intrinsic factors and host responses to tumor antigens. There are multidimensional communications between the microenvirons and GBM cells. GBM will progress and be resistant to therapy by communicating with and manipulating other cells in the brain environs (21). Microglial cells are the principal resident immune cells of the brain, and they play important roles in homeostatic functions in the brain, such as in defending against infectious pathogens, neurodegenerative diseases, or traumatic brain injury, but act favoring tumor proliferation in gliomas (22, 23). Monocytes also exist in the GBM microenvironment, and they will differentiate into macrophages when infiltrating tumors. Microglia, monocytes, and macrophages are together termed tumor-associated macrophages or myeloid cells (TAMs) (21). **Figure 1** shows the immunosuppressive microenvironment of GBM. GBM-associated macrophages and microglia secrete inhibitory cytokines, which decrease NK cell activity and T cell-mediated apoptosis and inhibit the binding and killing effects of T cells on antigen-presenting cells and GBM cells (24). This allows the tumor to escape the immune-killing effects of NK cells and T cells. Of the TAMs, monocytes and macrophages are recruited by cytokines, chemokines, or medical interventions. All TAMs can interact with GBM cells and play important roles in immunosuppression, neovascularization, and tumor proliferation (17, 25). Matrix metalloproteinases (MMPs)-2 is a crucial factor in facilitating GBM cell migration and invasion. MMP2 is released in a precursor form and cleaved to an activated state by MMP14, which is mainly secret by microglia to the GBM microenvironment (21). The increased levels of programmed cell death 1 ligand 1 (PD-L1) and indolamine 2,3-dioxygenase (IDO) expressed by GBM cells, and the limited self-presentation antigens caused by decreased major histocompatibility complex (MHC) expression are also part of the factors leading to immunosuppression in GBM. Microglial cells secrete TGFβ and IL-10, which downregulate the local myeloid and lymphoid immune cells and promote systemic immunosuppression (17). Pharmacological inhibition of cytokines such as TGFβ can partially reverse the immunosuppression of brain tumors (17, 26).

To date, many approaches have been explored to reverse local or systemic immunosuppression in GBM for improved immunotherapeutic efficacy, including oncolytic virotherapy (27), peptide-based therapeutic vaccination (28, 29), dendritic cell vaccination (30), chimeric antigen receptor (CAR) T-cell therapy (31), and immune-checkpoint inhibition. For example, antibodies directed against specific tumor fusion proteins or chimeric antigen receptor T cells (CAR T cells) provide specific and active immunity against specific cell types or tumor neoantigens, or checkpoint blockade inhibitors such as anti-PD-1/PD-L1 or anti-CTLA4 increase the overall activity of T cell responses, increase antitumor immunity (32). SurVaxM is a peptide mimic immunotherapeutic vaccine that was granted orphan drug designation for patients with GBM by the FDA in 2017. SurVaxM has a dual mechanism of action of stimulating T-cell immunity and antibody-directed survivin pathway inhibition, stimulating the immune system to kill survivin-containing tumor cells, and has demonstrated safety and tolerability in patients with malignant glioma in a phase I study (33). Combination therapy of SurVaxM with pembrolizumab in patients with first relapsed glioblastoma entered a phase II clinical trial (NCT04013672).

Another key obstacle to immunotherapy of GBM lies in the limited drug delivery across BBB, which consists of brain microvascular endothelial cells (BMEC), parietal cells, basement membrane, and astrocytes. Adjacent vascular endothelial cells form tight junctions and adherent junctions, effectively plugging the clefts between BMECs. Normally, only particles smaller than 1 nm can passively transmit through the pores, thus limiting drug delivery to the brain parenchyma (15). In the neurovascular unit of the BBB, immune cells such as perivascular macrophages and microglia can also influence BBB function and play important roles in regulating innate and adaptive immunity (34).

### Functional Nanomaterials for Glioblastoma Immunotherapy

**Figure 2:**
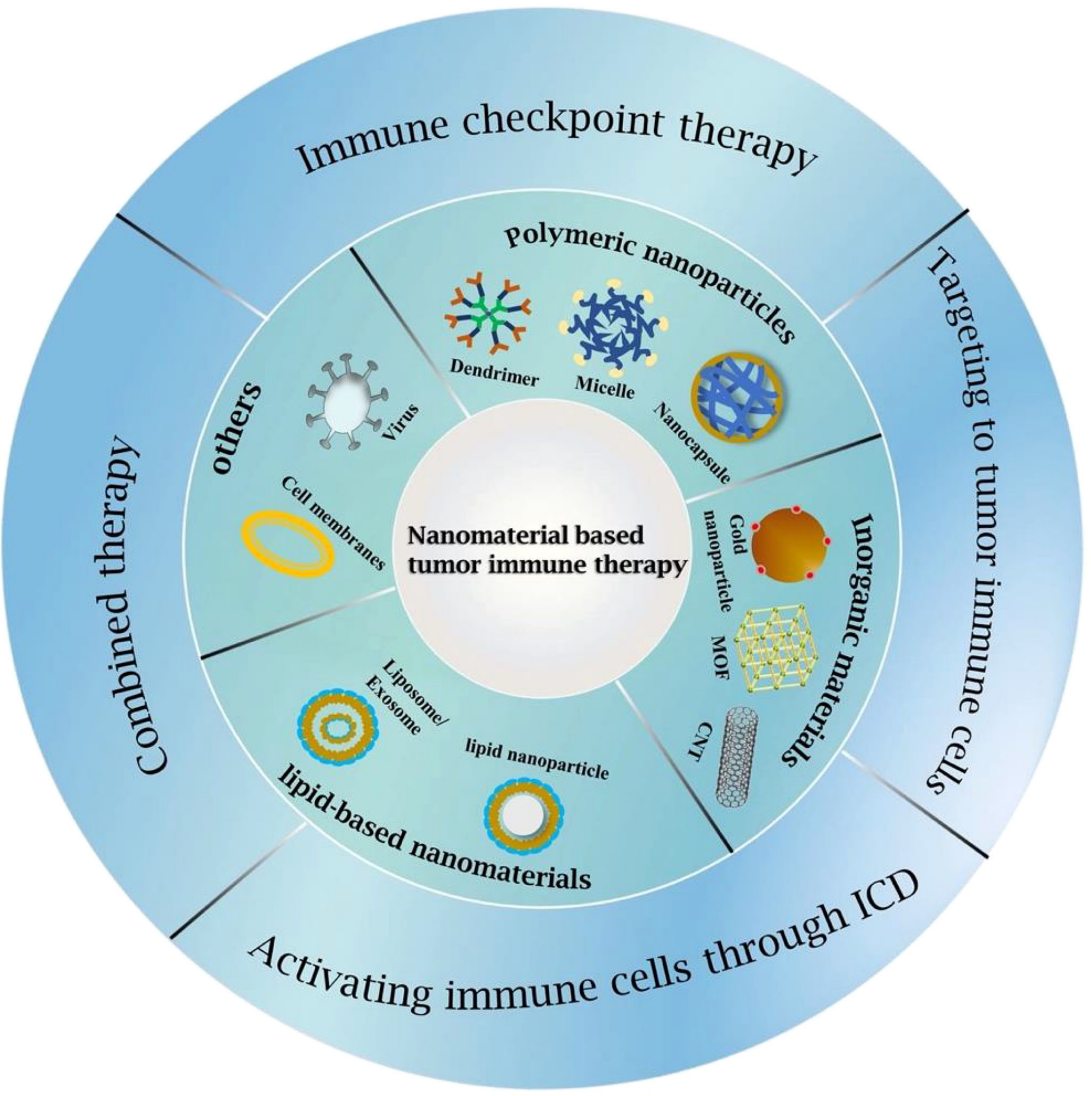
Classifications of GBM immunotherapy strategies and nanomaterials used for GBM immunotherapy.

To date, immunotherapies applied to GBM have achieved promising results in both preclinical and early clinical stages but failed to continue to exert their beneficial effects in later stages. The reasons for this are multiple: the high heterogeneity and plasticity of GBM make it prone to resistance to immunotherapy; the severe immunosuppressive GME, low mutation load and antigen presentation lead to poor response to GBM immunotherapy; the existence of BBB prevents most drugs from reaching and penetrating tumor tissue; and the drug itself has a short blood circulation time, which may cause problems such as systemic toxicity and autoimmune reactions. To address these challenges, nanomaterial-based drug delivery systems have been designed and developed. For the effective treatment of GBM, nanomaterials need to be efficient to cross the BBB, increase drug penetration and delivery to tumors, and have excellent stability and specific surface functional modifications. Several nanomaterials have been used as nanomedicines for clinical research and even marketing (35), including polymer materials, metal nanostructures, extracellular vesicles, liposomes, cell membranes, etc. In addition, administration routes such as nasal administration and intratumoral injection have also been developed to increase drug utilization and reduce drug loss. In this section, we will summarize the nanomedicines used in GBM immunotherapy in most recent years and classify them according to the pathway of eliciting immunity (**Figure 2**).

### Immune Checkpoint Therapy

Immune checkpoint molecules exist on the surface of both immune cells and tumor cells. Under physiological conditions, immune checkpoints are responsible for maintaining the balance of immune system responses and preventing excessive activation of immune cells, including stimulation and inhibition of both signaling pathways (36). Tumor cells subtly evade immune attack by dysregulation of immune checkpoint-related proteins. In the process of tumorigenesis and development, immune checkpoints have become one of the main reasons for immune tolerance. Immune checkpoint therapy will activate T cells to kill tumor cells through a series of pathways such as co-suppression or co-stimulatory signaling (37).

In tumor immunotherapy, the most studied immune checkpoints are the co-inhibitory molecules: programmed death receptor 1/programmed death-ligand 1 (PD1/PD-L1) and cytotoxic T-lymphocyte-associated protein 4 (CTLA-4) (38). Correspondingly developed immune checkpoint inhibitors (ICIs), such as anti-PD1/PD-L1 and anti-CTLA-4 can block the interaction between tumor cells that express immune checkpoint molecules and immune cells, thereby blocking the inhibition of tumor cells to immune cells. The application of ICIs is effective in the treatment of many tumors such as melanoma and non-small cell lung cancer but is ineffective in the treatment of GBM (39, 40). The BBB is one of the main factors limiting the therapeutic effect of ICIs. Because of their large molecular size, the concentration of mAbs that can be delivered to the brain is typically 1000 times lower than in blood without compromising biological activity (15). Galstyan et al. (41, 42) synthesized poly(β-L-malic acid) (PMLA)-based nano bioconjugates, capable of crossing the BBB *via* transferrin receptor (TfR)-mediated pinocytosis to target brain tumors. The covalent conjugate of anti-CTLA-4 or anti-PD-1 to PMLA (NICs) can stimulate T cell and macrophage responses in tumors. In the orthotopic mouse GL261 model, the number of CD8+ T cells in tumor tissues significantly increased after NIC treatment; the incidence of CD4+ FoxP3+ T cells was significantly decreased; tumor M1 macrophages were significantly increased; the systemic immune response was increased; serum interleukin levels were slightly elevated. Guo et al. (43) synthesized a conjugate of αPDL1 and p-hydroxybenzoic acid (pHA) (pHA-αPDL1), which can achieve BBB crossing of antibodies through dopamine receptor-mediated transcytosis. Compared with unmodified αPDL1, pHA-αPDL1 prolonged survival time and effectively inhibited tumor growth in the orthotopic GL261 model by activating glioma-infiltrating T cells and blocking PD-L1 on glioma cells. Wang et al. (44) used 2-Methacryloyloxyethyl phosphorylcholine (MPC), a choline analog, to prepare a BBB-penetrating copolymer. Then, the anti-PD-L1 was coupled to the copolymer *via* a pH-sensitive linker to form nanoparticles. In an orthotopic glioma model, such nanoparticles exhibited significantly improved BBB-penetrating ability *via* choline receptor-mediated transport after intravenous injection. Upon tumor accumulation, the anti-PD-L1 was released through the cleavage of the pH-sensitive linker in acidic GME. The levels of PD-L1-positive CD8+CD3+ T cells in glioma tissues after treatment were significantly increased, suggesting the PD-L1 blockade of tumor cells and the prevention of the immune escape of tumor cells. The number of CD8+CD3+ T-granzyme B [apoptotic effector secreted by cytotoxic T lymphocytes (ctl)] positive cells also increased, attributed to the enhanced immune response elicited by the efficient delivery and release of anti-PD-L1 to gliomas. Further results showed that the number of regulatory T cells (Treg) (CD4+CD3+Foxp3+) decreased, the immunosuppressive tumor microenvironment was relieved, and immune T cells induced antitumor immunity was activated.

### Targeting Tumor-Associated Immune Cells

GBM has a highly immunosuppressive tumor microenvironment, lacks immunogenicity, and most of them have a low tumor mutation burden (TMB). Unfortunately, both tumor cells and immune cells have been shown to contribute to this immunosuppressive phenotype (45). Among them, myeloid-derived suppressor cells (MDSCs) and tumor-associated macrophages (TAMs) are two types of cells that are mainly involved in suppressing antitumor immune responses in cancer patients, resulting in poor prognosis of GBM. Studies have shown that TAMs generally express an M2-like phenotype, inhibit the proliferation and activation of cytotoxic T cells and NK cells, and secrete numerous tumor-promoting cytokines and tumor growth factors to accelerate tumor growth, angiogenesis, progression and metastasis, and immunosuppression (46). MDSCs can inhibit the proliferation and activation of cytotoxic T cells and NK cells, and can also induce the polarization of macrophages from an inflammatory phenotype (M1) to an anti-inflammatory phenotype (M2), secreting a large number of tumor-promoting cytokines and tumor growth factors (47). Therefore, selectively targeting immune cells and reprogramming the tumor microenvironment have become an attractive therapeutic strategy to alleviate immunosuppression.

Hydroxy-terminated polyamidoamine dendrimers (PAMAM) are promising nanocarriers due to their small size, neutral surface charge, and high density of surface hydroxyl groups enabling them to cross the BBB and target activated microglia/macrophages (48, 49). Sharma et al. (50) conjugated rapamycin (Rapa) to PAMAM (D-Rapa) with a pH- cleavable linker. Rapa is a promising chemotherapy drug due to its inhibitory activity against mammalian targets of the mammalian target of rapamycin (mTOR) pathway. Rapa-mediated inhibition of the mTOR pathway is associated with the regulation of TAMs by promoting tumor angiogenesis and immunosuppression. Fluorescence-labled D-Rapa was highly specifically localized to TAMs 24h after intravenous injection and released from TAMs after 48h. In the orthotopic GL261 brain tumor model, D-Rapa improves knockdown of AKT phosphorylation compared to Rapa, while both treatments decrease expression of proliferative marker Ki67 and increase expression of apoptotic marker Caspase 3. Phosphorylation of colony-stimulating factor 1 (CSF-1) promotes the proliferation and transformation of macrophages into TAMs, and tumor cell invasion and metastasis. Clinical trials of various drugs targeting the CSF-1 pathway for cancer treatment are underway (NCT02829723, NCT02452424, NCT01349049). Liaw et al. (51) coupled the CSF-1R inhibitor BLZ945 to PAMAM (D-BLZ) through an acid-responsive linker. Compared with a free dose of BLZ945, a single systemic dose of D-BLZ can reduce the tumor-promoting expression of TAMs and promote CD8+ cytotoxic T cell infiltration, resulting in prolonged survival in mice with a reduced dose of BLZ945 required to achieve the same effect.

Glycosyl was recently discovered as a promising targeting ligand to tumor cells and TAMs for the increased metabolism in tumors (52–54). Sharma et al. (55) investigated the effect of surface modification of dendritic molecules such as glucose, mannose, and galactose on targeting glioblastoma. It was found that glucose modification significantly enhanced the targeting of TAMs and microglia by increasing brain penetration and cellular internalization, while galactose modification significantly targeted the mannose receptors (MRs) that are abundantly expressed on the TAMs. Mannose modification did not target TAMs and microglia but altered their accumulation kinetics in GBM. Liu group (56–58) investigated the tumor treating effects of mannose-modified liposomes, conventional naked liposomes, and polyethylene glycol liposomes. Mannose-modified liposomes promote the polarization of M0 and M2 towards the M1 phenotype by increasing the expression rate of CD86/CD206, and finally inhibiting the growth of G422 glioma. Chlorogenic acid (CHA) has been identified as a potent immunomodulator that promotes the polarization of TAMs from the M2 phenotype to the M1 phenotype. However, the rapid clearance and low tumor accumulation have affected the immunotherapeutic efficacy of CHA in clinical trials. Loading CHA into mannose-modified liposomes can achieve target delivery of CHA to TAMs, which can promote the polarization of TAMs from M2 phenotype to M1 phenotype by promoting the activation of STAT1 and inhibiting the activation of STAT6, thereby regulating the tumor microenvironment and inhibiting the growth of G422 glioma.

Mandruzzato group (59, 60) found that lauroyl-modified lipid nanocapsules (LNCs) could efficiently target mononuclear MDSCs and further investigated the effect of LNCs size and surface charge on cellular uptake. The results showed that neutral LNCs with 100 nm in size obtained the greatest uptake in mononuclear MDSCs, whereas positively charged 100-nm LNCs were more effective against macrophages and tumor cells. By tuning the size and charge of the material, it can be targeted to immunosuppressive cells, thus providing a new approach for utilizing nanosystems for antitumor therapy within the framework of immunotherapy.

### Activating Immune Cells Through Immunogenic Cell Death

Immunogenic cell death (ICD) induction strategy is a convenient way to achieve simultaneous activation of innate and adaptive immunity, by promoting the expression and exposure of stress-related molecules and the release of tumor antigens normally hidden within tumor cells (61, 62). ICD is accompanied by the exposure and release of numerous damage-associated molecular patterns (DAMPs), including calreticulin (CRT), heat shock proteins (HSPs), and high mobility group box 1 (HMGB1). As a source of antigen and adjuvant molecules, ICD activates antigen-presenting cells (APCs) and promotes anti-tumor immunity, providing a new direction for tumor immunotherapy. There are various methods to induce ICD, including chemotherapy, radiotherapy, and hyperthermia (HT) (63, 64). The ICD inducers are usually small-molecule compounds with a short half-life *in vivo*, easy clearance, low efficacy, and poor targeting. The delivery of small-molecule compounds by nanocarriers can prolong their half-life, and increase the drug efficacy and targeting efficiency.

Chen group (65, 66) investigated the role of doxorubicin-based nanodiamonds (Nano-DOX) in tumor therapy. Nano-DOX induces autophagy instead of apoptosis in GBM cells (GCs), and stimulates the release of antigens and DAMPs from GCs, resulting in enhanced activation of dendritic cells (DCs). Persano et al. (67) induced ICD of U87 glioblastoma cells through cubic iron oxide magnetic nanoparticles (IONC-GA-PEG) under the action of an external alternating magnetic field (AMF). Mild hyperthermia (MHT) mediated by IONC-GA-PEG induced up-regulation of several immunogenic molecules (CRT, HSP70, HSP90, and HMGB-1) and down-regulated immune “breaks” that promote immune escape (CD47 and PD-L1). In addition, MHT treatment was found to enhance NK cell recruitment at tumor sites, positively influence IL-2-activated NK cell degranulation and release IFN-γ, and may enhance GBM susceptibility to NK cell-mediated killing. Zhang et al. (68) synthesized bradykinin (BK) aggregation-induced-emission nanoparticles (BK@AIE NPs) with selective permeation of BBB and strong absorption in the near-infrared region (NIR). BK ligands can promote the activation of kinin B1 receptor (B1R), thereby enhancing the transport and accumulation inside the tumor. BK@AIE NPs have high photothermal conversion efficiency under 980 nm near-infrared laser irradiation, which is beneficial for the treatment of deep tumors. The therapeutic effect of BK@AIE NPs was evaluated in an orthotopic U87-MG tumor-bearing mouse model by intravenous injection. PTT-induced dead tumor cells release tumor-derived antigens that stimulate the host immune system, and the percentages of all cells infiltrating CD3+ T cells, CD4+ T cells, and CD8+ T cells in the tumor dramatically increased. As the immune system is activated, a series of representative cytokines interleukin-2 (IL-2), IL-10, IL-12, IL-1β, interferon γ (IFN-γ), tumor necrosis factor α (TNF-α) of T cells that regulate the immune response in the serum significantly increased, improved the anti-tumor immunity.

### Combined Therapy

GBM is a complex tumor involving various complex molecular pathways, genetic mutations, and tumor microenvironment. Despite extensive research, the treatment of GBM remains problematic. Poor drug delivery, tumor heterogeneity, and drug resistance pathways hinder the significant efficacy of monotherapy in GBM, which can easily lead to tumor recurrence (53). Therefore, combination therapy is considered a strategy to address this challenge. Ideally, drug combinations should leverage the strengths and weaknesses of each drug to improve efficacy, reduce toxicity, and overcome resistance.

Kadiyala et al. (69) designed a chemo-immuno combination therapy based on CpG (5’-C-phosphate-G-3’, a toll-like receptor 9 (TLR9) agonist) and docetaxel (DTX). The drugs were loaded on high-density lipoprotein nanodiscs (DTX-sHDL-CpG), which have a long circulation time in plasma. TLR9 ligands are expressed by most immune cells. CpG is a potent TLR9 agonist, causing activation of antigen-presenting cells (ie, macrophages and dendritic cells) in the GME with concomitant tumor antigen uptake. Activated dendritic cells migrate to draining lymph nodes, and present tumor antigens to CD8+ T cells, resulting in antitumor CD8+ T cell-mediated immunity. DTX enhances antitumor T cell responses to the tumor *via* ICD. In addition, radiotherapy is one of the treatment standards for GBM. DTX-sHDL-CpG combined with radiotherapy can cause tumor regression and long-term survival in 80% of GL26 mice, indicating the development of anti-GBM immune memory. Wang et al. (70) used perfluorocarbon (PFC) liquid-filled silica microshells to induce tissue damage through focused ultrasound to generate ICD and combined it with PD-1 blockade to induce a “hot” immune microenvironment and enhance immune checkpoint blockade against advanced tumors. Compared with monotherapy, combination therapy increased the proportion of CD45 leukocytes in the GME by more than 20 times, the proportion of CD8 cytotoxic T cells by more than 100 times, and the expression of IFNγ by more than 200 times, indicating the transition from “cold” to “hot” immune microenvironment. Li et al. (71) delivered nanosensitizers using neutrophils (NEs) to enhance GBM ultrasound/chemotherapy/immunotherapy. Immune checkpoint inhibitor (Anti-PD-1 antibody), paclitaxel (PTX), ZnGa_2_O_4_:Cr^3+^ (ZGO), and TiO_2_ are loaded in ROS-responsive liposomes to form ZGO@TiO2@ALP and delivered by NEs, which can penetrate the BBB and accumulated to GBM. After intravenous injection, ultrasound-triggered ZGO@TiO2@ALP could generate ROS and destruct liposomes to release PTX and anti-PD-1 antibodies to kill tumors and cause local inflammation, which in turn attracted more ZGO@TiO2@ALP-NEs migrate to the tumor site for enhanced and sustained treatment. The treatment improved the survival rate of the model GL261 mice from 0% to 40% and allowed long-term immune monitoring for tumor recurrence. Alghamri et al. (72) developed biocompatible NPs (SPNPs) composed of human serum albumin (HSA) and polyethylene glycol (PEG), functionalized with cell-penetrating peptide iRGD, capable of targeting tumors after systemic delivery. SPNPs loaded with CXCR4 inhibitor AMD3100 were able to block CXCR4 signaling in a GBM model, resulting in reduced infiltration of CXCR4+ MMDSCs into the GME. Blockade of CXCR4 sensitized GBM cells to radiation-induced ICD. SPNPs combined with radiotherapy elicited anti-GBM immune response, enhanced infiltration of CD3+ and CD8+ T cells, and T cells exhibited higher levels of expression of effector molecules (eg, Granzyme B, and IFN-γ), which eventually led to tumor disappearance in 60% of mice.

## Conclusion

Immunotherapy is one of the most promising ways to treat tumors. Various immunotherapy platforms are currently in clinical investigation, including various peptides, dendritic cells, heat shock protein vaccination strategies, excess T cell transfer, checkpoint blockade, monoclonal antibody, and cytokine therapy. However, the great potential of immunotherapy in GBM has been limited by several factors, including the severely suppressed immune microenvironment compared with other types of tumor, the limited drug delivery to the central nervous system, and safety issues such as autoimmune reactions, on-target, and off-target toxicity, cytokine storm, and dosing thresholds and so on. Most recent advances in understanding the physiology of the BBB, GBM microenvironment, and the immunosuppressive mechanism of GBM have provided us with great opportunities to develop effective immunotherapeutics against GBM. In addition, the emergence of nanotechnologies also provides a new development direction for the efficient targeted delivery of drugs to overcome physiological barriers and active targeting of specific cell populations, such as tumor cells/immune cell subsets. In GBM immunotherapy, rationally designed nanomaterials can directly reverse the immune status of the primary tumor by delivering ICIs or receptor agonists, inducing ICD, or others, and utilizing the potential of surrounding immune cells to prevent pre-metastatic niches formation and inhibition of tumor recurrence. Taken together, nanomaterial may uncage the great potential of immunotherapy in the treatment of GBM.

## Author Contributions

LT, MZ and CL conceived and designed the framework of this article. LT and MZ prepared the draft of the manuscript. CL reviewed and edited the manuscript. All the authors checked the article. All authors contributed to the article and approved the submitted version.

## Funding

This work was supported by the National Natural Science Foundation of China (No. 52073015), and Fundamental Research Funds for the Central Universities (No. ZY2006).

## Conflict of Interest

The authors declare that the research was conducted in the absence of any commercial or financial relationships that could be construed as a potential conflict of interest.

## Publisher’s Note

All claims expressed in this article are solely those of the authors and do not necessarily represent those of their affiliated organizations, or those of the publisher, the editors and the reviewers. Any product that may be evaluated in this article, or claim that may be made by its manufacturer, is not guaranteed or endorsed by the publisher.
